# Adult Onset Henoch-Schonlein Purpura and Intussusception: A Rare Presentation

**DOI:** 10.1155/2016/3957605

**Published:** 2016-09-26

**Authors:** Mridula Krishnan, Joseph Nahas

**Affiliations:** ^1^Department of Internal Medicine, Creighton University School of Medicine, 601 N 30th Street No. 5800, Omaha, NE 68131, USA; ^2^Department of Rheumatology, Creighton University School of Medicine, 601 N 30th Street No. 5800, Omaha, NE 68131, USA

## Abstract

We present an unusual case of a young 26-year-old male who was diagnosed with Henoch-Schonlein Purpura (HSP). Initial presentation was primarily mild gastrointestinal symptoms, which progressed to a life threatening intussusception and subsequently resolved with prompt glucocorticoid use rather than typical surgical intervention. Of importance, the patient's initial gastrointestinal symptoms without associated skin manifestations made the diagnosis difficult. In conclusion, it is important to recognize uncommon presentations of HSP as it may lead to life threatening complications and surgical intervention may be avoided with prompt treatment.

## 1. Introduction 

Henoch-Schonlein Purpura (HSP) is a leukocytoclastic vasculitis which involves small vessels with deposition of immune complexes containing IgA. It is characterized by the association of skin, joint, and gastrointestinal manifestations, which may occur in succession [1]. Significant morbidity can occur due to gastrointestinal and renal involvement. The incidence in adults has been estimated at 1.3 per 100,000, with a mean age at presentation of approximately 50 years [2, 3]. We will discuss a case where gastrointestinal symptoms preceded skin involvement leading to diagnostic difficulty.

## 2. Case History

A 26-year-old Caucasian male with no significant past medical history presented in the outpatient setting with constant, dull abdominal pain for a duration of six days, localized to the epigastric and left upper quadrant. He denied fever, chills, diarrhea, or blood in stool. The abdominal pain was associated with intermittent vomiting and decreased oral intake to solid foods. Abdominal ultrasound was unrevealing, and due to progressive pain, he was admitted for further evaluation. On the day of admission, he developed a new onset rash on his lower extremities. At the same time, he also developed new onset left-sided wrist pain and swelling.

On physical examination, he was anicteric. He was afebrile; pulse was 83 beats per minute, blood pressure was 134/100 mm Hg, respiratory rate was 35 per minute, and oxygen saturation was 95% on room air. His abdomen was diffusely tender to palpation with no distension, rigidity, or rebound tenderness. His liver and spleen were not enlarged and his bowel sounds were active. Examination of the skin revealed a palpable purpuric rash on his lower extremities bilaterally and on his back. His musculoskeletal examination revealed unilateral synovitis of the left wrist joint. The rest of the physical exam findings were unremarkable.

Laboratory studies revealed a normal white count of 10.8 k/*μ*L, hemoglobin of 17.1 g/dL, and a platelet count of 300,000 k/*μ*L. Basic metabolic studies were within normal limits, including his renal function. Erythrocyte sedimentation rate and C-reactive protein were elevated at 19 mm/hr and 50 mg/L, respectively. C3 and C4 complement values as well as IgA levels were within normal limits. Liver function tests were normal except that alanine transaminase (ALT) was mildly elevated at 107 *μ*/L. Hepatitis panel and anti-neutrophil cytoplasmic antibodies (ANCA) levels were negative. Urinalysis was negative for hematuria or proteinuria.

**Figure 1:**
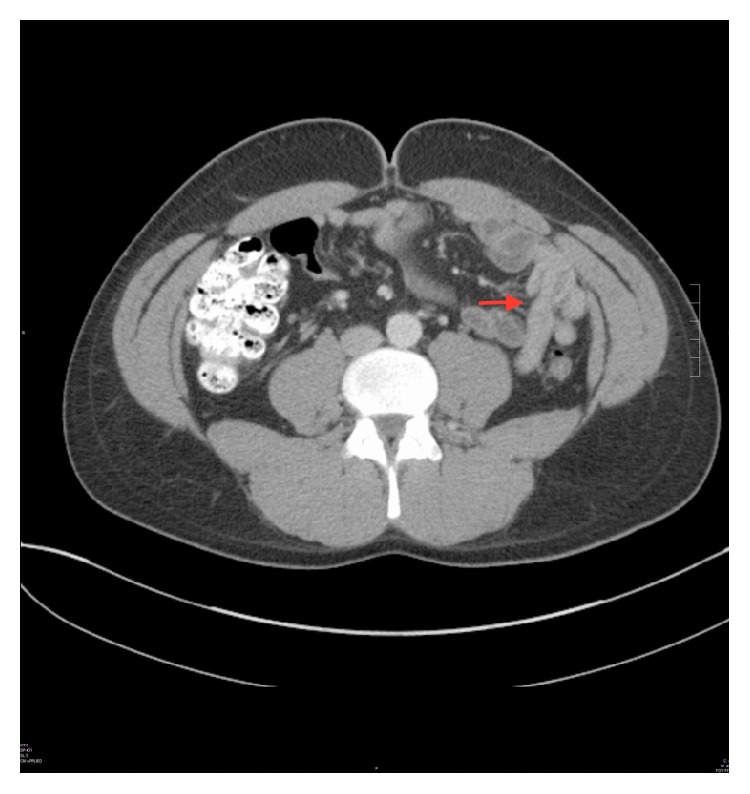
Focal small bowel intussusception in the jejunum.

A computed tomography (CT) of the abdomen with IV contrast was performed and it showed mild dilatation and thickening of the jejunum and a focal small bowel intussusception with left upper quadrant mesenteric lymphadenopathy and mild splenomegaly (Figure 1).

Given the CT findings of intussusception, general surgery was consulted for evaluation. The recommendation was to repeat a CT of the abdomen the following day and if the intussusception was still present, he would undergo a segmental resection. With the presumptive diagnosis of HSP based on clinical and lab values, rheumatology was consulted and the patient was started on intravenous methylprednisolone 60 mg once daily due to severe abdominal and joint pain.

By the next day, his abdominal pain had improved, he was able to tolerate oral intake, and a repeat CT scan done showed resolution of the intussusception. His joint pain also had improved. Throughout his hospital stay, he continued to improve and was switched to oral prednisone on discharge with rheumatology follow-up in one week. At his follow-up visits, his rash and arthritis had resolved and he did not have recurrence of his abdominal pain. Subsequently, he was tapered off of his prednisone without incident.

## 3. Discussion

Henoch-Schonlein Purpura (HSP) is a predominantly pediatric vasculitis. Ninety percent of cases occur between the ages of 3 and 15 years [4].

The diagnosis of HSP is based upon clinical manifestations of the disease [5]. The classical tetrad manifests with skin, joint, gastrointestinal, and renal involvement [1]. HSP in children has been extensively studied, and it is generally considered a self-limiting disorder [6]. In a large single center retrospective study performed in Northern Spain by Calvo-Río et al. with 417 patients (adults and children) with HSP, complete recovery was observed in most of the patients with a relapse rate of 31% [7].

In adults, HSP has been observed to have a higher frequency of systemic involvement. The outcome in adults was found to be similar to children with complete recovery from the disease in the majority of the patients [8].

Gastrointestinal symptoms occur in about one-half of HSP patients and range from mild symptoms to more significant findings such as intussusception, gastrointestinal hemorrhage, bowel ischemia, and perforation [9]. In a study involving HSP in the adult population, 24.1% of patients develop GI symptoms before the cutaneous rash [10]. Severe GI manifestations, such as intussusceptions, massive GI bleeding, and perforation, are much less common in adults [11, 12]. The most frequent of these GI complications is intussusception, which is the main reason for laparotomy, and it occurs at a rate of about 3% in HSP [5].

The main approach to the treatment of patients with intussusception associated with HSP is emergency laparotomy, although the evidence is limited to several case reports [13]. Pretreatment with glucocorticoids has been studied but is not established to have an effect on morbidity or early resolution of the illness; thus their use is controversial. Glucocorticoids have been shown in most studies to be beneficial in reducing the duration of abdominal pain. The pathophysiology of glucocorticoid use for gastrointestinal symptoms may be attributed to reduction in the edema of the intestinal wall. It has also been controversial whether they have an effect on prevention of intussusception development due to a limitation of studies [14, 15].

Also, glucocorticoid use in patients with severe abdominal pain or after an intussusception has occurred can mask the signs or symptoms of a perforation or worsening intussusception. Follow-up imaging studies in patients whose symptoms improve with glucocorticoids are needed to confirm the symptomatic resolution of an intussusception, and continued close monitoring is needed. Early recognition of HSP in adult patients whose abdominal pain occurs before the classic skin rash or joint pain can be difficult. In an epidemiologic study on the clinical spectrum of HSP in children from Northwest Spain, in 13 of them (16.7% of the children included in the study), abdominal pain was the initial manifestation, which preceded the onset of palpable purpura by 1 to 10 days (mean of 3.3 days) [16].

Specific to this case, intravenous glucocorticoids were used as the patient was unable to maintain oral intake and had severe abdominal pain requiring admission. The resolution of the patient's intussusception was likely due to the short duration of the disease, mild intussusception, and prompt treatment. The resolution of the mild intussusception and altered disease outcome with early intravenous glucocorticoid use, thus sparing the patient from undergoing a laparotomy and resection, was unique in our case.

## 4. Conclusion

Intussusception in HSP is very rarely seen in adults and the main modality of treatment for intussusception has been found to be a surgical approach. Studies have shown that glucocorticoid therapy shortens the duration of abdominal pain in patients with HSP but the role of glucocorticoids in the treatment of intussusception in HSP needs to be studied further [4].
